# 慢性移植物抗宿主病肺部病变诊治中国专家共识（2026年版）

**DOI:** 10.3760/cma.j.cn121090-20260520-00254

**Published:** 2026-08

**Authors:** 中华医学会血液学分会 中国医师学会呼吸病学分会 中国临床肿瘤学会白血病专业委员会 广东省基层医药学会免疫缺陷诊治专业委员会 广东省呼吸健康学会 广东省医学教育协会血液免疫诊断与治疗专业委员会

## Abstract

慢性移植物抗宿主病（cGVHD）肺部病变是异基因造血干细胞移植后极具临床意义的并发症，严重影响造血干细胞移植受者的生活质量和生存率。闭塞性细支气管炎综合征和间质性肺疾病是cGVHD肺部病变的两种主要分型，但因其起病隐匿，诊断困难且治疗方法有限，临床管理困难，患者预后较差。基于近年来cGVHD肺部病变研究新进展及逐渐增多的循证医学证据，本共识由国内多领域专家合作制定，为cGVHD肺部病变的早期识别、诊断、治疗和管理提出建议，旨在建立适用于我国临床应用的肺部cGVHD的标准化诊疗流程，以更好地指导临床实践，改善患者生活质量和生存率。

## Abstract (en)

Pulmonary chronic graft-versus-host disease（cGVHD）is a clinically significant complication after allogeneic hematopoietic stem cell transplantation, severely affecting the quality of life and survival rate of hematopoietic stem cell transplantation recipients. Bronchiolitis obliterans syndrome and interstitial lung diseases are the two main clinical entities. However, the insidious onset, diagnostic challenges, and limited therapeutic options of pulmonary cGVHD result in suboptimal clinical management and poor prognosis. Based on the latest developments in pulmonary cGVHD research and increasing evidence-based medicine data, this consensus was developed collaboratively by multidisciplinary experts domestically, providing recommendations for the early recognition, diagnosis, treatment, and management of pulmonary cGVHD, aiming to establish a standardized diagnostic and therapeutic workflow for pulmonary cGVHD applicable to clinical practice in China, to better guide clinical practice and improve patients' quality of life and survival rate.

慢性移植物抗宿主病（chronic graft-versus-host disease，cGVHD）是异基因造血干细胞移植（allogeneic hematopoietic stem cell transplantation，allo-HSCT）后的常见并发症，可累及全身多个器官，发生率为30％～70％[1]–[2]。cGVHD肺部病变是一种迟发性非感染性肺部并发症（late onset non-infectious pulmonary complication，LONIPC），其主要临床特征为进行性加重的胸闷、气促，伴进行性肺功能下降[3]–[4]。基于肺通气功能损害模式和胸部CT特征，以阻塞性通气功能障碍为主要表现的闭塞性细支气管炎综合征（bronchiolitis obliterans syndrome，BOS）是目前唯一公认的cGVHD肺部病变的表现形式[5]。临床研究显示，以限制性通气功能障碍为主要表现的间质性肺疾病（interstitial lung diseases，ILD）可能是肺部cGVHD的另一种表现形式[6]。尽管ILD目前尚未被公认为肺部cGVHD的表现形式，本共识结合既往研究数据及临床证据，提出此观点并呼吁临床医师对此类型cGVHD肺部病变保持高度警惕，在移植后出现肺部并发症的患者中积极评估cGVHD-ILD的可能性，因为cGVHD-ILD病情进展更迅速，死亡率更高，临床医师在临床诊疗中应充分重视。

cGVHD肺部病变早期可缺乏明显症状，部分allo-HSCT受者在尚无明显呼吸系统症状时已出现肺功能异常[7]。目前国际各个医疗中心报道的allo-HSCT后cGVHD肺部病变的发病率不尽一致，既往研究报道的发生率不一，BOS影响3％～10％的allo-HSCT受者[8]–[13]，当合并已确诊的cGVHD时，其发生率会上升至14％[12]–[14]。既往队列研究显示，BOS患者总体预后较差，5年生存率仅为10％～13％[13]–[18]。但近年来随着对BOS认识的提高及肺功能监测的规范化，更多患者在早期即被识别并接受临床干预，其长期生存率已明显改善，近年研究显示其5年生存率约70％[17]–[18]。ILD的发病率较BOS低，3年累积发病率为2％～5％[4],[17],[19]，但由于疾病早期诊断十分困难，其发病率可能被低估。

cGVHD肺部病变，无论是BOS还是ILD，严重影响造血干细胞移植（HSCT）后患者的生活质量和生存率。同时，cGVHD肺部病变具有起病隐匿、早期临床表现缺乏特异性、诊断高度依赖肺功能及影像学动态评估并需与感染、药物相关肺损伤及其他LONIPC鉴别等特点，其诊断评估体系、动态监测策略及治疗管理均具有较强的专科特征，现有综合性cGVHD指南难以充分覆盖临床实践中的关键问题。因此，重视cGVHD肺部病变的诊治具有重要意义。目前尚缺乏可供临床广泛参考的针对cGVHD肺部病变的标准化治疗方案。基于此，本共识将在现有临床观察和实践经验的基础上，参考国内外相关指南和共识并结合新近研究成果，制定cGVHD肺部病变的相关诊疗建议，旨在为进一步规范化诊治cGVHD肺部病变提供参考。

一、共识制定方法

本共识由中华医学会血液学分会、中国医师学会呼吸病学分会、中国临床肿瘤学会白血病专业委员会、广东省基层医药学会免疫缺陷诊治专业委员会、广东省呼吸健康学会、广东省医学教育协会血液免疫诊断与治疗专业委员会共同发起，共识专家组由血液科、呼吸与危重症医学科、感染科、重症医学科、药剂科等国内多个领域共70多位专家组成。编写组于2025年3月组建并召开工作会议，按照人群、干预、对照和结局（population, intervention, comparison, outcome，PICO）原则拟定共识纲要和关键问题，以“allo-HSCT” “chronic graft-versus-host disease” “pulmonary cGVHD” “bronchiolitis obliterans syndrome” “BOS” “interstitial lung disease” “ILD” “organizing pneumonia” “nonspecific interstitial pneumonia” “pleuroparenchymal fibroelastosis” “异基因造血干细胞移植”“慢性移植物抗宿主病”“闭塞性细支气管炎综合征”“间质性肺疾病”“机化性肺炎”“非特异性间质性肺炎”“胸膜肺弹力纤维增生症”等为关键词，检索PubMed、Web of Science、Embase、The Cochrane Library、中国知网、万方数据库和维普网数据库等中外数据库，检索范围为建库至2026年4月。纳入研究类型为中英文系统评价或荟萃分析、随机对照研究、队列研究、病例对照研究、病例报告；同时对已纳入的指南或荟萃分析的参考文献进行追溯，以补充证据。本共识采用2009版牛津大学循证医学中心证据分级与推荐强度标准对每个临床问题的证据质量和推荐强度进行分级（表1）。

**表1:** 英国牛津大学循证医学中心证据分级与推荐强度分级标准

推荐强度	证据级别	描述
A	1a	随机对照试验（RCT）的系统评价
	1b	结果可信区间小的RCT
	1c	显示“全或无效应”的任何证据
B	2a	队列研究的系统评价
	2b	单个的队列研究（包括低质量的RCT，如失访率>20％）
	2c	基于患者结局的研究
	3a	病例对照研究的系统评价
	3b	单个病例对照研究
C	4	病例系列报告、低质量队列研究和低质量病例对照研究
D	5	基于未经验证的专家意见或评论或基础实验

1. 目标人群与使用者：本共识适用目标人群为接受allo‑HSCT后存在cGVHD肺部病变发生风险、临床疑似及已确诊cGVHD肺部病变的患者，覆盖BOS、ILD两种临床表型。本共识供从事HSCT及移植后患者管理的血液科、呼吸与危重症医学科、感染科、重症医学科、儿科、药学、肺移植专科及其他相关专业医务人员参考使用，适用于国内开展allo‑HSCT随访及cGVHD肺部病变诊疗工作的各级医疗机构。

2. 参与共识制订的专家分组和职责：共识制订工作由发起单位统筹。执笔专家负责共识框架、证据汇总、文本及推荐意见草案的撰写；秘书组负责文献检索、资料整理、会议组织、意见收集及文稿协调；特别顾问提供学术指导；参与共识审阅和讨论的专家负责审阅证据及推荐意见草案，并结合多学科临床实践提出修改意见。各组成员及其单位见文末“本共识制订专家委员会名单”。

3. 临床问题的遴选、推荐意见形成和共识达成：临床问题的遴选和确定遵循临床重要性、实践差异性和可回答性原则。编写组结合现有指南与共识、相关循证证据及我国临床实践，按照PICO原则初步提出临床问题，经共识专家讨论、反馈和修订后确定纳入范围及关键结局。推荐意见草案以证据质量和推荐强度分级结果为基础，综合考虑干预的获益与风险、患者适用性、资源可及性及我国临床实践的可实施性形成，并在专家审阅后根据反馈进行修改。

4. 共识判定方法：推荐意见采用改良德尔菲法进行匿名审阅与表决；共进行两轮，预先设定赞同率≥70％为达成共识。未达到阈值的推荐意见根据专家意见修改后进入下一轮审阅或表决，直至达成共识或决定不纳入。

二、cGVHD肺部病变的发生机制、危险因素及临床特征

1. 发生机制：cGVHD肺部病变是全身性cGVHD的器官特异性表现，与cGVHD的发生机制一致，是一个多阶段、多因素参与的过程，主要涉及免疫调节失衡、炎症反应以及组织损伤和纤维化等病理过程[20]–[22]。组织损伤刺激炎症因子释放，引起慢性持续性炎症反应，导致胸腺损伤及B细胞和T细胞等免疫活性细胞功能失调，持续的免疫炎症反应可导致组织损伤、组织纤维化和器官功能障碍。这些病理反应既是一个连续的过程，也可同时存在，相互重叠和相互强化。另外，肺部通过气道与外界直接相通，allo-HSCT受者作为免疫抑制人群，持续暴露于微生物环境，使感染成为加速疾病演进的核心因素，这一特性显著区别于其他部位的cGVHD。

2. 危险因素：目前关于cGVHD肺部病变的危险因素的认知仍在不断提升，不同研究结果也存在一定差异。其发生可能与以下因素有关：①受者及移植相关因素：清髓预处理方案（含白消安方案）、高龄、移植前存在基础肺疾病、女性供者或供受者性别不匹配、ABO血型不合等[23]–[25]；②移植相关并发症：既往发生急性GVHD（aGVHD）（尤其是Ⅱ～Ⅳ级）或肺外cGVHD[23]–[24]；③呼吸道感染：呼吸道感染也是诱发cGVHD肺部病变的高危因素，包括病毒、细菌、真菌及非典型病原体[4],[26]；④其他：胃食管反流病、微误吸、空气污染物、既往胸部放疗等[27]。

3. 临床特征：cGVHD肺部病变的临床表现缺乏特异性，主要表现为慢性、进行性加重的呼吸困难、运动耐量下降及喘息。肺部体征与临床分型密切相关，不同表型之间存在一定差异。BOS起病隐匿，早期可无明显症状，随着疾病进展逐渐出现气流受限相关表现，呈现阻塞性肺气肿的临床体征；而ILD多在起病初期表现间质性肺炎相关临床特征。

BOS的临床表现包括慢性干咳、劳力性呼吸困难、运动耐量下降、喘息。临床症状通常在中重度患者中出现，其中20％～30％表现为明显的气流下降，呼吸衰竭患者表现为唇、指甲发绀等缺氧表现；严重BOS晚期患者可能出现二氧化碳潴留临床表现；肺部叩诊过清音，听诊呼吸音减弱，或可伴有呼气相干啰音。BOS患者20％可合并胸腔漏气综合征，表现为气胸、纵隔气肿及皮下气肿[28]。另外，BOS患者易合并肺部感染[29]，若临床发现HSCT后患者反复发生肺感染，应考虑鉴别BOS。

ILD的临床表现具有异质性，且与其病理亚型密切相关。患者临床表现多以低氧为主，主要表现为活动后气促、干咳，气促较BOS更加明显[17]。听诊可闻及velcro啰音，严重患者有杵状指、发绀，合并肺动脉高压患者可有下肢水肿、颈静脉怒张等右心衰竭表现。

三、cGVHD肺部病变的诊断

**临床问题1：**cGVHD-BOS和cGVHD-ILD的诊断标准分别有哪些？

**推荐意见1.1：**cGVHD肺部病变早期缺乏特异性表现。建议移植后定期随访及自我监测，并于移植前（进入移植仓前）3～6个月且病情相对稳定阶段进行肺功能及活动耐量等检测，以该阶段记录到的最佳肺功能及活动耐量等参数作为个体化基线参考。若出现活动耐量下降、不明原因消瘦、乏力、纳差及情绪改变等，应警惕cGVHD肺部受累并积极开展诊断筛查（推荐强度C，证据等级4）。

**推荐意见1.2：**cGVHD-BOS和ILD的诊断主要依据临床症状、体征、肺功能、胸部影像学及实验室检查等，也需排除肺部感染及急性肺损伤等其他疾病，建议必要时进行专科会诊或多学科会诊明确诊断。cGVHD-BOS的诊断标准及严重程度分级标准见表2和表3（推荐强度A，证据等级1a）。cGVHD-ILD诊断标准见表4（推荐强度B，证据等级2c）。

**表2:** cGVHD-BOS的诊断标准（参照NIH 2014[30]）

allo-HSCT患者满足以下4条标准即可诊断：
1. FEV_1_/FVC<0.7或<预测值的第5百分位数（90％*CI*下限）。
2. FEV_1_<75％pred，在2年内下降≥10％；且在吸入β_2_受体激动剂（如沙丁胺醇）后FEV_1_增加量<200 ml，改善率<12％。
3. 根据临床症状进行高分辨率CT、微生物培养（鼻拭子、咽拭子、痰培养、支气管肺泡灌洗液）等检查，除外呼吸道感染。
4. BOS的3个相关支持证据（满足其中1项即可）：①存在其他部位cGVHD表现；②呼气相高分辨率CT显示空气潴留征、小气道壁增厚或气道扩张；③肺功能检查有空气滞留的证据：RV>120％pred或RV/TLC比值>90％*CI*。

**注** cGVHD：慢性移植物抗宿主病；BOS：闭塞性细支气管炎综合征；NIH：美国国立卫生研究院；allo-HSCT：异基因造血干细胞移植；FEV_1_：第1秒用力呼气量；FVC：用力肺活量；pred：预计值；RV：残气量；TLC：肺总量。对于无法进行肺功能检查的患者，可结合症状评分（如改良英国医学研究委员会呼吸困难评分、慢性阻塞性肺疾病评估测试评分）、胸部影像学表现及氧合情况进行综合评估

**表3:** cGVHD-BOS的严重程度分级标准[13],[30]

分级	第1秒用力呼气量（FEV_1_）
轻度	60％～79％pred
中度	40％～59％pred
重度	≤39％pred

**注** cGVHD：慢性移植物抗宿主病；BOS：闭塞性细支气管炎综合征；pred：预计值

**表4:** cGVHD-ILD的诊断标准[19],[31]

allo-HSCT患者满足以下4条标准考虑诊断：
1. 高分辨率CT显示肺泡和（或）间质性异常；
2. 呼吸道样本［支气管肺泡灌洗液和（或）鼻腔分泌物和（或）痰液］中未检出有临床意义的致病病原体，结合微生物学检测结果及临床评估综合判定，排除活动性感染；
3. TLC<80％基线值^a^，且FEV_1_/FVC>0.7；若同时合并BOS时，TLC<80％且FEV_1_/FVC<0.7；
4. DLCO<80％基线值^a^

**注** cGVHD：慢性移植物抗宿主病；ILD：间质性肺疾病；allo-HSCT：异基因造血干细胞移植；TLC：肺总量；FEV_1_：第1秒用力呼气量；FVC：用力肺活量；BOS：闭塞性细支气管炎综合征；DLCO：一氧化碳弥散量；^a^基线值为造血干细胞移植前的最佳肺功能

目前尚无cGVHD-ILD严重程度分级标准，临床需结合多维度指标综合评估。cGVHD-ILD的严重程度建议围绕以下关键因素综合评估，通常表现为在过去1年内，符合以下3条标准中至少2条：

（1）呼吸道症状恶化；

（2）有疾病进展的生理证据，包括：①用力肺活量占预计值百分比（FVC％pred）绝对值下降≥5％；②校正血红蛋白后的一氧化碳弥散量占预计值百分比（DLCO％pred）绝对值下降≥10％；

（3）一种或多种疾病进展的放射学证据（典型影像学表现见图1）。

**图1:**
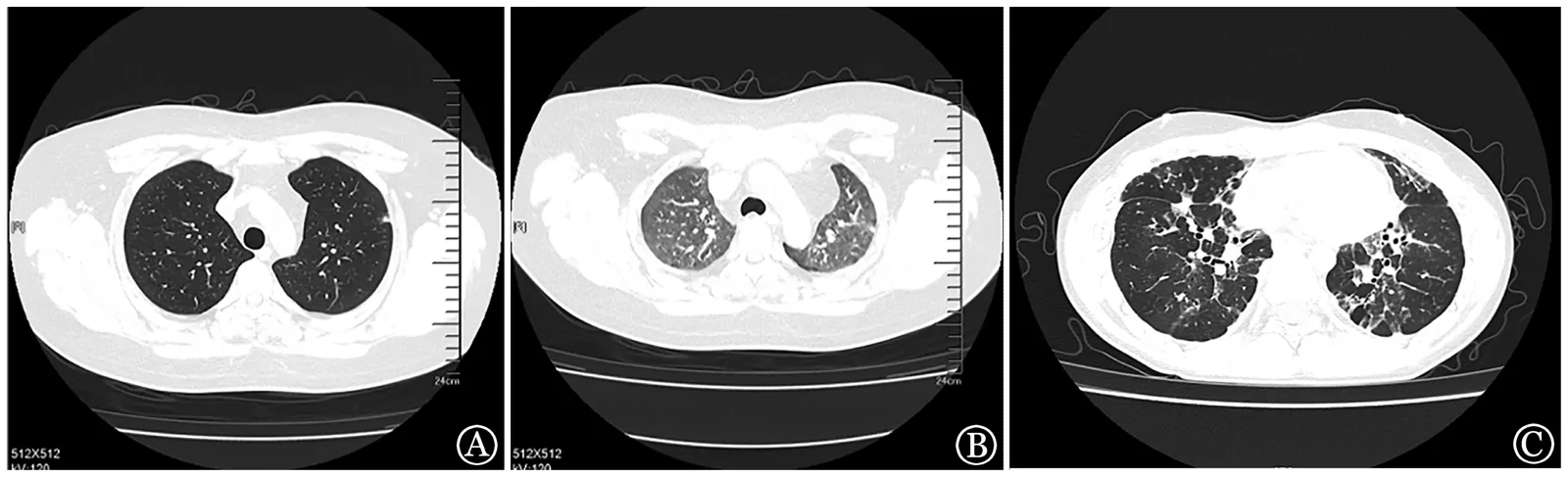
慢性移植物抗宿主病（cGVHD）患者肺部病变的影像学表现 **注** A：闭塞性细支气管炎综合征（BOS）患者吸气相CT，主要显示马赛克灌注征：肺实质密度呈斑片状、拼图样不均匀，低密度区（病变区）与相对高密度区（正常或代偿区）交替分布；B：BOS患者呼气相CT，呈现空气潴留这一关键征象：病变区域的肺组织在呼气时密度不升高、体积不缩小，持续保持低密度，与正常呼气相肺密度均匀增高形成鲜明对比；C：间质性肺疾病患者高分辨率CT表现，双肺网状阴影，以胸膜下为著，散在磨玻璃影，伴胸膜增厚

四、cGVHD肺部病变的鉴别诊断

在cGVHD肺部病变的诊断中，排除其他病因非常重要，包括肺部感染及肺部非感染性疾病：①肺部感染：allo-HSCT后一旦出现新的呼吸道症状，应首先考虑感染因素，并对患者进行全面病原体检测，包括影像学［高分辨率CT（HRCT）］、血清学检查及支气管肺泡灌洗等。若患者处于肺部感染状态，首先积极控制感染，再进一步明确BOS诊断。需注意，BOS也常同时合并感染，且感染也可诱发BOS。②肺部非感染性疾病：包括隐源性机化性肺炎（COP）、急性间质性肺炎（AIP）及aGVHD等，可通过临床表现、肺功能及影像学特征综合判断。

**临床问题2：**cGVHD肺部病变的诊断流程如何？

**推荐意见2：**cGVHD肺部病变的诊断流程图见图2。另注意，在allo-HSCT患者中，肺部感染与cGVHD肺部病变可能并存。当患者同时具备感染及cGVHD危险因素时，必须通过严格排查病原体，结合影像学动态变化及肺功能演变进行综合评估（推荐强度C，证据等级4）。

**图2:**
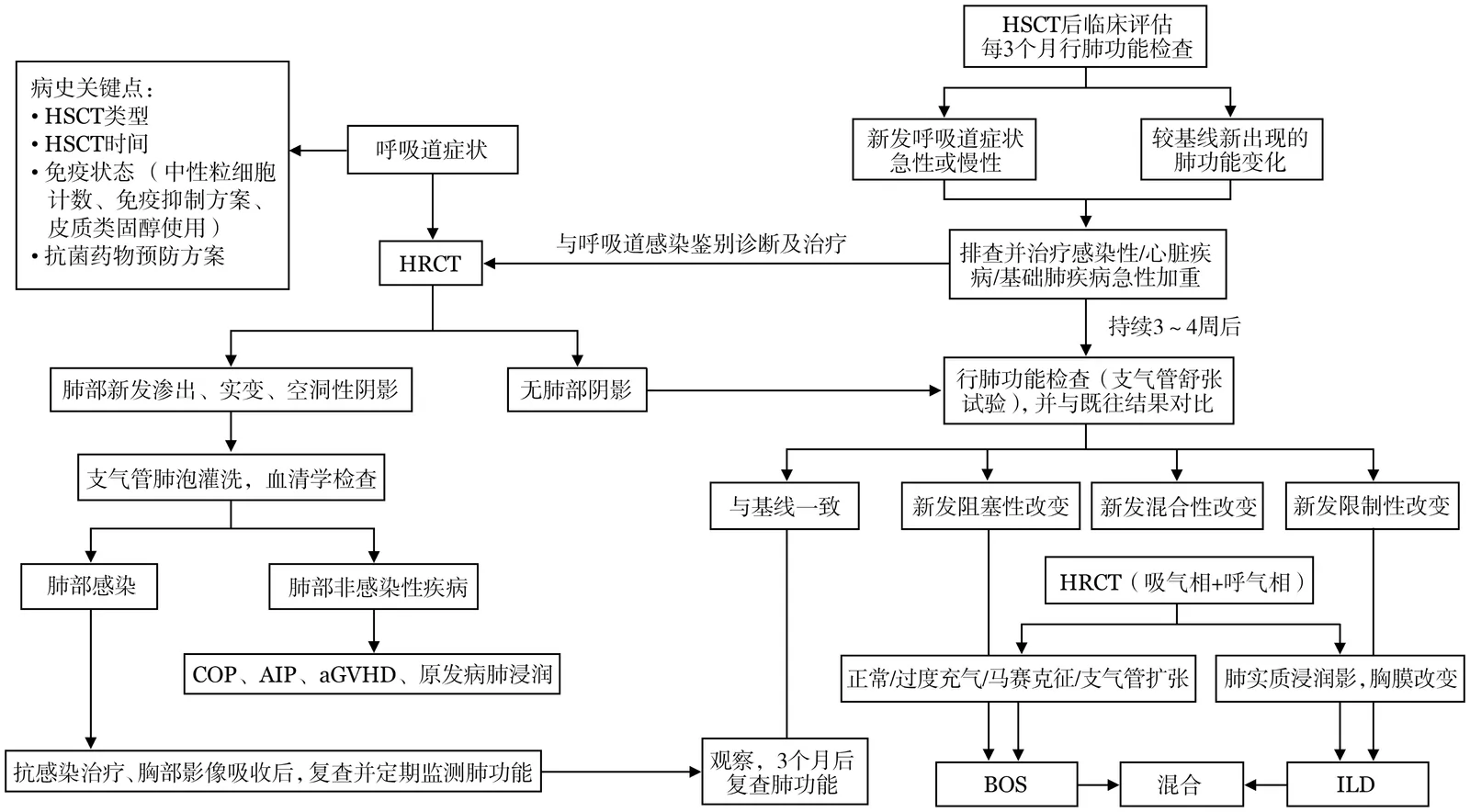
慢性移植物抗宿主病（cGVHD）肺部病变的诊断流程图 **注** 当鉴别感染与cGVHD肺部病变时，若两者诊断均符合，则考虑cGVHD肺部病变合并肺部感染；当cGVHD肺部病变表现为ILD时，常伴肺外cGVHD表现，应在排除感染的同时，系统评估全身受累情况。HSCT：造血干细胞移植；HRCT：高分辨率CT；COP：隐源性机化性肺炎；AIP：急性间质性肺炎；aGVHD：急性移植物抗宿主病；BOS：闭塞性细支气管炎综合征；ILD：间质性肺疾病

**临床问题3：**allo-HSCT后，为尽早诊断cGVHD肺部病变，肺功能检查应多久进行一次？对于确诊cGVHD肺部病变的患者，肺功能检查应多久进行一次？HRCT评估应多久进行一次？

**推荐意见3：**

①对于allo-HSCT受者，为实现cGVHD肺部病变的早期识别，建议在移植后出院前至少完成1次肺功能检查；在移植后1～3个月内再次进行肺功能检查。无肺部症状的受者之后每3～6个月进行1次肺功能检查。②若肺部CT出现异常征象，或有新发呼吸道症状，强烈推荐尽快启动肺功能检查，并根据评估结果及时启动相应临床干预措施；治疗后复查肺功能，之后根据肺功能转归情况调整监测频率，通常建议至少每3～6个月进行1次肺功能检查。③对于已确诊cGVHD肺部病变的患者，建议至少每3个月进行1次肺功能检查；对于病情活动或合并感染等不稳定状态患者，建议增加监测频率（如每2周1次或每月1次）。在长期随访过程中，应根据个体病情变化对监测频率进行动态调整。④推荐在诊断及有指征（如肺功能下降或出现呼吸道症状）时进行双相（吸气相+呼气相）HRCT扫描，以排除其他病因（推荐强度B，证据等级2b）。

监测重点：对于cGVHD肺部病变患者，建议进行完整的肺功能检查。对于cGVHD-BOS患者，监测重点为肺通气功能指标，包括FEV_1_、FEV_1_/用力呼气量（forced vital capacity，FVC）、残气量（residual volume，RV）、RV/肺总量（total lung capacity，TLC）指标；对于cGVHD-ILD患者，应同时评估肺通气功能及弥散功能，重点关注FVC、TLC、一氧化碳弥散量（diffusing capacity of the lung for carbon monoxide，DLCO）指标。

证据总结：研究表明[32]，FEV_1_在诊断BOS前6个月内即可出现进行性下降，且可能于临床症状出现前。因此，定期进行肺功能检查对于早期识别cGVHD肺部病变至关重要[33]。国际指南建议，无症状的allo-HSCT受者在移植后第1年每3个月检查1次，第2年每6个月检查1次，此后至少5年根据临床情况每年复查1次[7],[34]。另有研究表明[8]，BOS诊断后前3个月内FEV_1_可能出现快速下降。因此，在BOS诊断后早期阶段建议增加肺功能监测频率（如前3个月每月1次）[29]；对于临床状况不稳定、出现新症状或其他器官发生GVHD、发生呼吸道感染或治疗方案发生变化的患者，亦建议适当增加检查频率[35]；长期随访过程中，肺功能监测应结合治疗方案进行个体化调整，以评估早期或延迟疗效；出现病情恶化、新发症状或并发其他疾病时，可考虑适当增加监测频率（如每月1次），以便及时识别病情进展并指导治疗；当病情达到临床稳定状态时，可逐步降低监测频率至每3～6个月1次[7]。

胸部CT检查是BOS诊断的重要辅助工具。研究证实[36]，LONIPC的CT特征分为闭塞性细支气管炎（bronchiolitis obliterans，BO）和不可分类的间质性肺炎，提示CT特征可能有助于放射科医师对LONIPC做出早期诊断，以指导早期治疗。多项研究表明[37]–[38]，HRCT可发现大部分BOS患者的异常表现，但早期BOS患者也可基本正常或只显示非特异性异常。在诊断及有指征（如肺功能下降或出现呼吸道症状）时进行双相（吸气相+呼气相）胸部HRCT扫描，有助于排除其他病因。

**临床问题4：**何种情况下需要进行支气管镜检查？

**推荐意见4：**对于存在肺功能下降，肺部影像学提示异常［如小气道扩张、空气潴留征、马赛克衰减征、肺泡和（或）间质异常等］，肺部疾病诊断不明确的患者，建议行支气管镜检查以协助诊断；推荐行支气管镜肺泡灌洗（BAL）排除感染性病因；必要时可在有经验的移植中心谨慎考虑行肺活检以明确诊断，并根据病变部位、患者状态及预期诊断获益选择适宜的取材方式。临床应用过程中需格外谨慎，应充分告知患者可能出现的严重并发症，包括气胸、纵隔气肿、持续性气漏甚至死亡，避免有创操作加重病情（推荐强度C，证据等级4）。

证据总结：研究显示[39]，对于HSCT后发生BOS的患者，超细支气管镜可观察到多种气道闭塞表现，如膜性闭塞、瘢痕增生性闭塞、支气管环状狭窄或黏液增多，提示支气管镜检查可能对BOS的诊断有一定意义。临床实践文章建议[40]，在BOS诊断过程中需排除感染性病因，BAL可用于明确是否合并活动性感染。研究证实[41]，对于疑似BO患者，肺活检有助于明确allo-HSCT后的肺部病变性质。当临床、肺功能及影像学评估后仍无法明确诊断，肺活检可作为补充诊断手段，有助于鉴别allo-HSCT后不同类型肺部病变，并为临床决策提供参考。美国国立卫生研究院（NIH）共识中提到，肺活检证实的BO可直接作为cGVHD肺部病变的诊断依据，BO的典型组织病理学表现为细支气管管腔部分或完全闭塞，伴黏膜下及管周纤维化、肉芽组织增生等改变[42]。ILD缺乏特异性的组织病理学表现，可呈现机化性肺炎（organizing pneumonia，OP）、非特异性间质性肺炎（nonspecific interstitial pneumonia，NSIP）及胸膜肺弹力纤维增生症（pleuroparenchymal fibroelastosis，PPFE）等不同组织病理学类型，需结合临床、肺功能及影像学综合判断[17]。需注意的是，肺活检可能导致气胸、纵隔气肿、持续性气漏综合征甚至死亡等严重并发症，临床应用要格外谨慎[13]。

五、cGVHD肺部病变的治疗

1. cGVHD肺部病变的治疗原则：当前针对cGVHD肺部病变的治疗尚未形成统一标准的方案，临床实践中推荐多维度的综合干预策略，治疗方案汇总见表5。治疗的核心目标是控制肺部组织损伤、改善肺功能、减少治疗相关不良反应[43]。各临床中心应结合患者具体情况实施个体化的治疗选择，需考虑以下核心因素[13],[20]–[21],[35],[44]：

**表5:** 慢性移植物抗宿主病（cGVHD）肺部病变的治疗方案

方案	治疗建议	推荐强度及证据等级
初始治疗	
糖皮质激素联合或不联合CNI	推荐糖皮质激素联合或不联合CNI作为cGVHD肺部病变一线治疗的标准方案，推荐剂量见《慢性移植物抗宿主病诊断与治疗中国专家共识（2024年版）》[21]；在治疗过程中，糖皮质激素可进行短期疗效评估（一般不超过3周），若无明确临床获益，应及时停用；若有临床获益，建议根据临床情况动态调整，优先逐步减停激素，其他免疫抑制剂每2～4周减量1次，每3～9个月减停1种。免疫抑制剂治疗疗程应个体化制定，中位维持时间建议1～3年。西罗莫司可被考虑作为联合免疫抑制治疗的潜在选择。	B；2b
ICS联合或不联合LABA	强烈推荐cGVHD-BOS患者在常规免疫抑制治疗方案基础上加用ICS，可联用或不联用LABA。	A；1b
联合或不联合FAM方案	推荐cGVHD-BOS患者在常规免疫抑制治疗方案基础上加用FAM方案进行治疗，推荐剂量：吸入性氟替卡松440 µg每日2次；阿奇霉素250 mg每周3次；孟鲁司特10 mg每晚1次。应定期监测接受FAM方案治疗患者的症状、不良事件和疾病进展情况以判断是否继续使用。	B；2b
进展治疗	
贝舒地尔	推荐cGVHD肺部病变患者在常规免疫抑制方案基础上加用贝舒地尔，基线存在血细胞减少或感染风险患者优先选择贝舒地尔。推荐剂量：200 mg，每日1次。	B；2b
芦可替尼	推荐cGVHD肺部病变患者在常规免疫抑制治疗方案基础上加用芦可替尼，尤其是合并难治性cGVHD的患者，但应谨慎用于有（复发性）感染和血细胞减少的患者。建议起始剂量5～10 mg，每日2次，使用中需定期监测血象、感染风险、肝肾功能等。	B；2b
伊布替尼	不推荐cGVHD肺部病变患者在常规免疫抑制治疗方案基础上加用伊布替尼。	D；5
其他药物	cGVHD肺部病变若呈进展性纤维化表型，有条件的临床中心可根据患者个体情况酌情合用吡非尼酮或尼达尼布治疗。	B；2b
MSC	对于初始治疗无效的患者，推荐根据临床经验和患者病情进行MSC输注，但尚不存在广泛应用的条件。	B；2b
肺移植	对于经药物治疗无效且呼吸衰竭仍进行性加重的终末期cGVHD肺部病变的患者，建议转诊至肺移植专科中心，在严格评估个体状况及移植相关风险后，将肺移植作为挽救生命的治疗选择。	B；2c

**注** CNI：钙调磷酸酶抑制剂；ICS：吸入性糖皮质激素；LABA：长效β_2_受体激动剂；BOS：闭塞性细支气管炎综合征；FAM方案：氟替卡松、阿奇霉素和（或）孟鲁司特；MSC：间充质干细胞

（1）cGVHD肺部病变的临床表型及有无合并其他靶器官受累：如BOS以阻塞性通气功能障碍为主、ILD以限制性通气功能障碍及弥散功能下降为核心表现的差异；是否合并肺外其他部位cGVHD（如皮肤、消化道等）；

（2）疾病临床评估：如肺功能检查（FEV_1_、FVC等指标）的动态变化、严重程度评分［如第1秒用力呼气量占预计值百分比（FEV_1_％pred）、肺部症状评分］等；此外，细胞因子水平、免疫细胞亚群特征等指标也可用于cGVHD肺部病变的预后判断及疗效评估；

（3）患者整体身体状况：包括基础疾病、体能状态及对治疗的耐受性等；

（4）治疗反应评估：根据肺功能检查评估患者对治疗的反应，根据病情变化及治疗效果调整治疗方案；治疗过程中涉及药物剂量调整、药物更换或停用时，应由相关专科医师综合评估后实施，以保证治疗方案的连续性与安全性；

（5）治疗cGVHD药物不良反应：如感染、肝功能异常、血细胞减少、骨髓抑制、代谢异常等；

（6）还应考虑合并症管理、药物可及性、患者依从性等因素。

在常规治疗方案效果不佳时，应积极鼓励患者进入临床试验，通过探索新型靶向药物、细胞治疗或联合干预策略，持续优化治疗手段，改善患者的临床结局与生存质量。

目前缺乏前瞻性数据明确cGVHD肺部病变治疗的最佳疗程。cGVHD肺部病变免疫抑制治疗疗程需个体化制定，在保证治疗安全性与耐受性的基础上，以达成临床稳定（症状控制、肺功能及影像学稳定）为核心目标。建议治疗后每3～6个月进行1次全面评估（症状、肺功能、影像学等），并根据评估结果调整治疗方案和监测频率。

2. cGVHD肺部病变的初始治疗方案：

**临床问题5：**cGVHD肺部病变的一线用药及疗程如何？

**推荐意见5：**推荐糖皮质激素联合或不联合钙调磷酸酶抑制剂（CNI）作为cGVHD肺部病变一线治疗的标准方案，推荐剂量见《慢性移植物抗宿主病诊断与治疗中国专家共识（2024年版）》[21]；在治疗过程中，糖皮质激素可进行短期疗效评估（一般不超过3周），若无明确临床获益，应及时停用；若有临床获益，建议根据临床情况动态调整，优先逐步减停激素，其他免疫抑制剂每2～4周减量1次，3～9个月时间减停1种。免疫抑制剂的治疗疗程应个体化制定，中位维持时间建议1～3年。西罗莫司可被考虑作为联合免疫抑制治疗的潜在选择（推荐强度B，证据等级2b）。

证据总结：多项共识提出[13],[35],[42]，cGVHD肺部病变的一线治疗药物通常包括皮质类固醇，如泼尼松（剂量1 mg·kg^−1^·d^−1^）。此外，考虑到长期应用糖皮质激素不良反应多，部分BOS患者对激素应答有限，可与其他免疫抑制药物联合应用以提高反应率并减少糖皮质激素用量，如CNI类药物包括环孢素A、他克莫司[45]。临床研究已证实，联合CNI类药物可以减少糖皮质激素用量，治疗反应率为20％～40％[46]。目前尚缺乏确定cGVHD肺部病变全身糖皮质激素最佳疗程的高质量证据[35]。国际共识将激素难治cGVHD定义为泼尼松≥1 mg·kg^−1^·d^−1^，治疗1～2周后疾病仍持续进展[47]。提示临床实践中通常在短期内即可对激素治疗反应进行初步评估。有研究表示[48]，联合西罗莫司对cGVHD进行初始治疗是一种可行的替代方案。

**临床问题6：**对于cGVHD-BOS患者，除常规免疫抑制治疗方案外，是否应加用吸入性糖皮质激素（ICS）联合或不联合长效β_2_受体激动剂（LABA）？

**推荐意见6：**强烈推荐cGVHD-BOS患者在常规免疫抑制治疗方案基础上加用ICS，可联用或不联用LABA（推荐强度A，证据等级1b）。

证据总结：近年，在常规免疫抑制治疗基础上联合应用ICS和LABA可改善患者临床症状得到验证。布地奈德/福莫特罗可抑制免疫介导的肺部炎症损伤，快速缓解气道痉挛，改善气流受限，从而缓解cGVHD肺部病变的症状[49]。多项研究证实[50]–[51]，联合吸入布地奈德、福莫特罗治疗可显著改善BOS患者的FEV_1_和FVC，可有效缓解呼吸系统症状，且该疗效维持稳定6个月，无严重不良反应。

**临床问题7：**对于cGVHD-BOS患者，除常规免疫抑制治疗方案外，是否应加用氟替卡松、阿奇霉素和（或）孟鲁司特（FAM方案）进行治疗？

**推荐意见7：**推荐cGVHD-BOS患者在常规免疫抑制治疗方案基础上加用FAM方案进行治疗，推荐剂量：吸入性氟替卡松440 µg每日2次；阿奇霉素250 mg每周3次；孟鲁司特10 mg每晚1次。应定期监测接受FAM方案治疗患者的症状、不良事件和疾病进展情况以判断是否继续使用（推荐强度B，证据等级2b）。

证据总结：临床研究显示，FAM方案可稳定成人cGVHD-BOS的肺功能[52]，联合FAM方案治疗3个月后，94％的患者病情稳定，延缓了肺功能下降速度，且该方案耐受性良好，糖皮质激素用量也明显减少，显著改善了患者的生活质量。然而，阿奇霉素对BOS长期肺功能改善的疗效尚不明确。有荟萃分析显示[53]，阿奇霉素可在短期至中期（≤24周）显著改善移植后BOS患者的肺功能，但其长期疗效呈减弱趋势，可能与长期用药产生耐药性有关。另外，长期使用阿奇霉素治疗BOS可能与血液病复发风险和后续新发肿瘤风险增加有关[54]–[55]。值得注意的是，长期使用阿奇霉素治疗应定期监测肝功能[35],[56]。接受FAM方案治疗的患者需定期监测症状、不良事件及疾病进展，以评估治疗反应及是否需要继续或调整用药方案。

3. cGVHD肺部病变进展后的治疗方案：对于糖皮质激素依赖或难治性cGVHD肺部病变患者，可选择贝舒地尔、芦可替尼作为后续治疗选择。贝舒地尔具有免疫调节及抗纤维化作用，已有研究显示可使BOS患者获益，并可能对ILD具有潜在治疗价值；芦可替尼以抗炎作用为主，BOS患者同样被证实可获益。不建议使用伊布替尼治疗。条件允许的中心可在多学科评估和临床研究框架下，个体化考虑使用阿巴西普、艾克利单抗、小剂量IL-2受体拮抗剂、利妥昔单抗、伊马替尼等；若呈进展性纤维化表型，可在呼吸专科联合评估下酌情合用吡非尼酮/尼达尼布。总体而言，贝舒地尔与芦可替尼是目前cGVHD肺部病变的主要二线治疗选择，并在规范化慢病管理及临床研究框架内实施个体化治疗。

**临床问题8：**对于cGVHD肺部病变患者，除常规免疫抑制治疗方案外，是否加用Rho关联卷曲螺旋蛋白激酶2（ROCK2）抑制剂贝舒地尔治疗？

**推荐意见8：**推荐cGVHD肺部病变患者在常规免疫抑制方案基础上加用贝舒地尔，对于基线存在血细胞减少或感染风险的患者，优先选择贝舒地尔。推荐剂量：200 mg，每日1次（推荐强度B，证据等级2b）。

证据总结：贝舒地尔是一种选择性ROCK2抑制剂，具有抗炎和抗纤维化双重作用机制。贝舒地尔可重建免疫稳态，减少促炎细胞因子产生并促进调节性T细胞（Treg）生成[57]–[58]，还可直接作用于成纤维细胞ROCK2靶点，阻断其分化并诱导肌成纤维细胞凋亡，减少胶原沉积，发挥直接抗纤维化作用，同时通过作用于炎症阶段间接减少纤维化形成[59]。两项贝舒地尔治疗cGVHD-BOS的前瞻性研究汇总分析显示[60]，综合肺功能与症状评估的最佳总体缓解率（overall response rate，ORR）为37％，Lee症状量表（LSS）肺部评分也明显改善，达到最佳临床反应的中位时间约为7个月；另外，ORR与基线NIH肺评分存在明显相关性，基线NIH肺评分为1、2、3分患者的ORR分别为53％、26％、0。此外，在针对cGVHD的Meta分析亚组中，肺部受累患者也观察到获益[61]。提示尽早启动贝舒地尔治疗的cGVHD-BOS患者的治疗反应和预后更好。另有研究表明[62]，对于基线存在血细胞减少的患者可优先使用贝舒地尔。

**临床问题9：**对于cGVHD肺部病变患者，除常规免疫抑制治疗方案外，是否应加用芦可替尼治疗？

**推荐意见9：**推荐cGVHD肺部病变患者在常规免疫抑制治疗方案的基础上加用芦可替尼，尤其是合并难治性cGVHD的患者，但应谨慎用于有（复发性）感染和血细胞减少的患者。建议起始剂量5～10 mg，每日2次，使用中需定期监测血象、感染风险、肝肾功能等（推荐强度B，证据等级2b）。

证据总结：芦可替尼是一种选择性JAK 1/2抑制剂，可抑制效应性Ｔ细胞和促炎细胞因子生成[63]。临床研究表明[64]，芦可替尼可以改善allo-HSCT后BOS患者的肺功能，达到最佳临床反应的中位时间约为10个月，3级及以上的严重不良事件发生率约为35％，其中血液学严重不良事件发生率约为10％。安全性方面，有荟萃分析提示，芦可替尼常见的血液学不良事件为血小板减少、贫血和白细胞减少，非血液学不良事件为感染性并发症[65]。另有研究表明，芦可替尼应谨慎用于有（复发性）感染和血细胞减少病史的患者[35]。

**临床问题10：**对于cGVHD肺部病变患者，除常规免疫抑制治疗方案外，是否应加用伊布替尼治疗？

**推荐意见10：**不推荐cGVHD肺部病变患者在常规免疫抑制治疗方案基础上加用伊布替尼（推荐强度D，证据等级5）。

证据总结：伊布替尼可以通过抑制B细胞和T细胞的活化发挥免疫抑制作用，从而缓解cGVHD[57],[66]。但目前尚无研究专门探讨伊布替尼治疗cGVHD-BOS的疗效，通常不建议加用伊布替尼，其有效性及安全性还需更多的高质量、大规模临床研究证实。

**临床问题11：**对于cGVHD肺部病变患者，除常规免疫抑制治疗方案外，是否应加用抗纤维化药物（吡非尼酮、尼达尼布）进行治疗？

**推荐意见11：**cGVHD肺部病变若呈进展性纤维化表型，有条件的临床中心可根据患者个体情况酌情合用吡非尼酮或尼达尼布治疗（推荐强度B，证据等级2b）。

证据总结：吡非尼酮和尼达尼布是近年应用于临床治疗的新型抗纤维化药物[13]。目前有关吡非尼酮、尼达尼布在cGVHD肺部病变中的治疗证据较少。有临床研究显示[67]，吡非尼酮治疗后1年内FEV_1_％pred改善了约7个百分点，SF-36身体功能评分及LSS症状评分也均有改善，但不良事件发生率较高（81.8％），最常见的不良反应为恶心、呕吐、疲劳及体重减轻。另有探索性研究结果显示[68]，低剂量芦可替尼联合尼达尼布治疗重症BOS可获得58.3％的缓解率；安全性方面，主要不良反应为2级血胆红素升高和3～4级血小板减少。尽管针对cGVHD-ILD的直接证据有限，吡非尼酮和尼达尼布在多种其他类型的ILD中已展现出明确的疗效和可接受的安全性[69]–[70]。

综上所述，免疫抑制治疗在cGVHD肺部病变的管理中具有核心地位。治疗过程应保证足够疗程，用药期间应注意严密监测血象与感染；对于疗效有限或反复感染的患者，应在权衡病情控制与感染风险后，考虑减少或停用部分免疫抑制剂，以降低感染风险[71]–[72]。

**临床问题12：**对于cGVHD肺部病变患者，是否应该接受间充质干细胞（mesenchymal stem cells，MSC）治疗？

**推荐意见12：**对于初始治疗无效的患者，推荐可根据临床经验和患者病情进行MSC输注，但尚不存在广泛应用的条件（推荐强度B，证据等级2b）。

证据总结：MSC作为一种细胞治疗载体，可通过促进Treg增殖活化，调控Th1/Th2比例发挥免疫调节作用，也可通过上调CD27^+^记忆B细胞数量、降低血清B细胞激活因子（BAFF）水平和促进B细胞表面BAFF受体表达诱导免疫耐受，从而改善GVHD[73]–[76]。临床研究显示[77]，MSC治疗BOS 3个月后，MSC组缓解率显著高于非MSC组（71％对44％），且76％的患者类固醇剂量减少≥50％，显著优于非MSC组（46％）。另有研究证实，MSC可改善BOS肺功能[78]，MSC治疗6个月后，FEV_1_、FEV_1_/FVC及血气指标（动脉血氧分压、脉搏血氧饱和度和肺泡-动脉氧分压差）均较基线显著升高。

**临床问题13：**cGVHD肺部病变患者是否应该接受肺移植治疗？

**推荐意见13：**对于经药物治疗无效且呼吸衰竭仍进行性加重的终末期cGVHD肺部病变患者，建议转诊至肺移植专科中心，在严格评估个体状况及移植相关风险后，可将肺移植作为挽救生命的治疗选择（推荐强度B，证据等级2c）。

证据总结：多项临床研究证实[79]–[86]，对于药物治疗无效，持续存在严重气流受限并伴肺功能进行性降低的BOS患者，肺移植为其提供了长期存活的机会[13]。有回顾性研究显示[81]，cGVHD-BOS患者在肺移植术后1年生存率可达90％，5年生存率可达75％。提示肺移植可能是治疗终末期cGVHD肺部病变的一种有效治疗手段。肺移植术前全面及个体化评估并分析其预后及不利因素是至关重要的。

六、确诊cGVHD肺部病变患者的随访及疗效评估

**临床问题14：**cGVHD肺部病变患者如何进行随访？

**推荐意见14：**对于cGVHD肺部病变患者，随访应遵循多维度、个体化、长期性原则，综合运用临床症状、肺功能、影像学及生活质量评估工具。强烈推荐cGVHD肺部病变患者每3～6个月进行1次随访，具体频率取决于疾病严重程度及肺功能下降速度（推荐强度C，证据等级4）。

随访内容具体阐述：每3～6个月进行1次综合评估[7]：①临床症状评估：咳嗽、咳痰、呼吸困难（活动后/静息时）、喘息、胸闷等症状的性质、频率和严重程度；②肺功能检查：包括肺通气功能检查和弥散功能检查，是监测cGVHD肺部病变最敏感、最客观的指标；③肺部影像学检查：包括评估肺部病变的性质、范围、程度变化；④生活质量与功能评估：可根据患者具体情况动态选择评估工具。6分钟步行试验（6MWT）可客观评估患者的运动耐力；SF-36量表可全面评估疾病对患者日常生活和身心状态的影响；对于合并系统性cGVHD的肺部受累患者，可使用LSS症状评分评估全身症状负担。

**临床问题15：**cGVHD肺部病变患者如何进行疗效评估？

**推荐意见15：**对于cGVHD-BOS患者，强烈推荐采用FEV_1_变化较基线变化率评估疗效（表6）（推荐强度A，证据等级1a）。由于BOS的疾病进展与临床难治，评估为疾病稳定（stable disease，SD）及以上状态则可认为治疗有效。对于cGVHD-ILD患者，建议采用综合评估法评估疗效（表7）（推荐强度C，证据等级4）。

**表6:** cGVHD-BOS疗效评估表

疗效	评估标准[87]
完全缓解（CR）	治疗后FEV_1_％pred≥80％
部分缓解（PR）	初始FEV_1_％pred<70％，治疗后FEV_1_％pred增加10％及以上，但尚未达CR
疾病稳定（SD）	治疗后FEV_1_％pred±10％
疾病进展（PD）	治疗后FEV_1_％pred下降≥10％

**注** cGVHD：慢性移植物抗宿主病；BOS：闭塞性细支气管炎综合征；FEV_1_％pred：第1秒用力呼气量占预计值百分比

**表7:** cGVHD-ILD疗效评估表

疗效	评估标准[88]–[89]
完全缓解（CR）	同时满足以下所有条件：①肺功能：FVC％pred较基线增加≥10％或恢复正常（≥80％ pred）；DLCO％pred较基线增加15％或恢复正常（≥80％ pred）。②影像学HRCT：肺部磨玻璃影、实变等渗出性病变完全吸收；纤维化病变（网格影、牵拉性支气管扩张等）稳定或无明确进展。③临床症状：咳嗽、呼吸困难等呼吸道症状完全消失或恢复至基线水平。
部分缓解（PR）	满足以下条件之一，但未达到CR标准：①肺功能：FVC％pred较基线增加≥5％且<10％和（或）DLCO％pred较基线增加≥10％且<15％。②影像学HRCT：渗出性病变部分吸收，纤维化病变稳定。③临床症状：呼吸道症状较前明显改善。
疾病稳定（SD）	满足以下所有条件：①肺功能：FVC％pred较基线变化在−5％至+5％之间；DLCO％pred较基线变化在−10％至+10％之间。②影像学HRCT：病变总体稳定，无明显吸收或进展。③临床症状：稳定，无显著恶化。
疾病进展（PD）	满足以下条件之一：①肺功能：过去1年内FVC％pred较基线下降≥5％和（或）DLCO％pred较基线下降≥10％。②影像学HRCT：出现新的渗出性病变，或原有病变明确进展，包括牵拉性支气管/细支气管扩张范围或程度增加、新发磨玻璃影伴牵拉性支气管扩张、新发细网状影、网状影范围扩大或纹理变粗、新发或范围增加的蜂窝状改变、肺叶体积损失增加。③临床症状：呼吸困难等症状持续且明确加重。

**注** cGVHD：慢性移植物抗宿主病；ILD：间质性肺疾病；FVC％pred：用力肺活量占预计值百分比；pred：预计值；DLCO：一氧化碳弥散量；HRCT：高分辨率CT

七、cGVHD肺部病变的抢先干预

**临床问题16：**已存在肺功能下降尚未达到BOS诊断标准的患者是否需要进行抢先干预？如何进行抢先干预？

**推荐意见16.1：**对于allo-HSCT后患者，若在定期随访监测中出现以下情况：①无论是否存在临床症状，存在cGVHD肺部病变高危因素（危险因素参见前文“二”中的“2. 危险因素”）；②肺功能较个体化基线明显下降，在关注FEV_1_变化的同时，建议结合其他反映小气道功能的指标进行综合评估；③肺部影像学提示新发异常阴影，应提高对早期BOS的警惕，强烈推荐启动cGVHD肺部病变的抢先干预评估与治疗（推荐强度B，证据等级2b）。

**推荐意见16.2：**对于存在较高cGVHD肺部病变发病风险及提示早期BOS或BOS-0p阶段（2020 NIH将BOS-0p阶段定义为已存在FEV_1_下降但尚不符合BOS诊断标准[13]）的allo-HSCT患者，建议密切监测肺功能变化，防控感染并尽早启动治疗。在抢先干预药物选择时，应充分考虑药物相互作用及药物不良反应包括骨髓移植风险、对免疫重建的影响及长期耐受性等。建议可抢先启动一线治疗方案，若治疗效果不佳可考虑应用贝舒地尔等二线治疗药物，并密切监测肺功能（推荐强度C，证据等级4）。

证据总结：研究发现，FEV_1_在确诊cGVHD肺部病变前6个月或更短时间内，甚至在出现临床症状前就可能迅速下降，通过定期监测肺功能，在FEV_1_较基线下降阶段及时干预，可改善患者预后[32]。国内外指南/共识均强调了早期干预的重要性，并指出若患者存在明确的cGVHD肺部病变高危因素，应提高筛查肺功能的频率，监测肺功能进展，尽量控制危险因素[13]。最大呼气中期流量（MMEF）可反映小气道功能状态，其早期下降与后续BOS发生风险相关，可作为BOS早期预警的辅助指标[33]–[34]。BOS-0p阶段患者肺部小气道和间质已存在早期损伤，任何诱因（如感染）都有可能会加速进展，因此在BOS-0p阶段进行早期干预是延缓或预防其进展为典型BOS的有力窗口期[90]。肺部感染和cGVHD肺部病变往往同时或相继发生，且感染可诱发cGVHD症状加重，是allo-HSCT后BOS患者的主要死亡原因，给cGVHD的诊断和治疗带来了一定难度，应积极控制感染，预防疾病进展。

目前cGVHD肺部病变的标准化抢先干预方案尚未完全确立，但临床中已存在一些被认可的抢先干预措施，核心目标仍是最大限度阻止病变进展为不可逆的cGVHD-BOS。2025 NIH将贝舒地尔列为具有潜力的抢先干预候选药物[91]。目前已有临床试验在探索贝舒地尔作为抢先干预措施的作用（NCT05922761）[91]。DeFilipp等[60]对贝舒地尔治疗BOS的汇总分析说明，基线NIH肺评分与治疗反应密切相关，提示对于BOS-0p阶段的allo-HSCT受者，尽早识别并启动贝舒地尔治疗可能会改善临床结局。另有研究表明，阿奇霉素也可考虑用于抢先干预，BOS-0p阶段的肺移植受者接受阿奇霉素抢先干预后[92]，44％的患者FEV_1_改善≥10％，提示在BOS-0p阶段启动阿奇霉素治疗获益更佳。但其安全性仍存在争议，有研究表明，早期使用阿奇霉素可能与血液病复发风险和后续新发肿瘤的风险增加有关[54]。

八、感染的预防与康复

**临床问题17：**cGVHD肺部病变患者如何加强感染预防？

**推荐意见17：**cGVHD肺部病变的患者可以通过接种流感病毒（灭活型）疫苗、肺炎链球菌疫苗、新型冠状病毒（灭活型）疫苗、呼吸道合胞病毒疫苗等预防感染；在免疫抑制治疗期间，基于个体风险因素，考虑使用抗病毒和抗菌药物预防；若患者有严重感染且IgG水平<400 mg/dl，应考虑使用免疫球蛋白（推荐强度B，证据等级2b）。

证据总结：国内外共识均鼓励cGVHD肺部病变患者针对流感病毒、肺炎链球菌、新型冠状病毒和呼吸道合胞病毒等进行疫苗接种，预防呼吸道感染以降低与免疫抑制相关的风险[7],[35]。有指南建议，HSCT患者应终身每年接种流感病毒疫苗（优先选择灭活型疫苗），且患者的家人、密切接触者和医护人员也应每年接种流感病毒疫苗；建议allo-HSCT患者接种3剂肺炎链球菌结合疫苗，间隔1个月，若发生GVHD，进行第4剂接种；建议allo-HSCT患者接受新型冠状病毒疫苗接种，建议优先接种mRNA疫苗[93]；对于cGVHD患者，在免疫抑制治疗期间，基于个体风险因素，可以进行抗病毒和抗菌药物预防[94]–[95]；对于患有严重感染且IgG水平<400 mg/dl的cGVHD患者，可考虑使用免疫球蛋白[35]。

**临床问题18：**对于肺部结构破坏的cGVHD肺部病变患者，合并真菌、铜绿假单胞菌定植，是否需要去定植治疗？

**推荐意见18：**建议根据具体情况，个体化采用针对性抗生素或抗真菌药物雾化吸入予去定植治疗。如铜绿假单胞菌定植，可选择雾化吸入的抗生素包括妥布霉素吸入剂、多黏菌素吸入剂、阿米卡星脂质体吸入剂等；而曲霉菌或念珠菌等真菌的定植，推荐采用两性霉素B雾化吸入（推荐强度C，证据等级4）。

证据总结：有系统评价研究表明[96]，铜绿假单胞菌在HSCT中的患病率为3％～100％，抗生素中妥布霉素吸入剂耐药性最低，其次是阿米卡星脂质体吸入剂及多黏菌素吸入剂等。在临床实践中，推荐抗生素的雾化吸入采用振动筛孔雾化器以提高药物在肺部的沉积率[97]。侵袭性真菌感染是HSCT患者发病和死亡的重要原因，曲霉菌和念珠菌是最常见的致病菌[98]。有研究表明[99]，两性霉素B雾化吸入治疗对于真菌定植具有良好的去定植作用且无明显不良反应。

**临床问题19：**对于真菌感染高风险的cGVHD肺部病变患者，可以采取哪些抗真菌预防用药？

**推荐意见19：**对于近期接受强化免疫抑制治疗且真菌感染高风险或有真菌定植的cGVHD肺部病变患者，推荐应用抗真菌药物进行预防，抗真菌药物可以选择三唑类药物如泊沙康唑、艾沙康唑、伏立康唑等，或棘白菌素类药物如卡泊芬净或多烯类药物如两性霉素B等全身治疗；同时可以考虑两性霉素B局部雾化吸入。具体疗程根据患者服用免疫抑制剂的强度、机体的免疫状态及真菌感染的高危因素综合决定。具体方案及疗程根据患者真菌感染风险、气道检查、呼吸道真菌培养、不良反应等因素进行综合评估（推荐强度C，证据等级4）。

证据总结：国内外指南及共识均指出[98],[100]，对于HSCT受者，可考虑在GVHD/全身免疫抑制期间进行预防性抗真菌治疗，预防性治疗可显著降低侵袭性真菌病（IFD）的发生率，并可降低真菌感染相关死亡率。可选择药物包括泊沙康唑、艾沙康唑和伏立康唑等三唑类药物，卡泊芬净等棘白菌素类药物和两性霉素B。预防性治疗时需综合考虑感染风险、感染进展速度、严重程度及患者具体病情进行个体化治疗[98],[101]–[103]。

**临床问题20：**对于接受免疫抑制治疗的cGVHD肺部病变患者，针对巨细胞病毒（CMV）可以采取哪些预防用药？

**推荐意见20：**对于接受免疫抑制治疗的cGVHD肺部病变患者高危人群，推荐采用更昔洛韦、缬更昔洛韦或来特莫韦予以预防；无法耐受长期普遍预防的患者，应该严密监测血CMV-DNA并优化用药方案（推荐强度C，证据等级4）。

证据总结：CMV感染是allo-HSCT后常见的感染并发症之一，也是导致患者预后不良的主要原因之一[104]。cGVHD肺部病变及其免疫抑制治疗是CMV感染的重要危险因素[105]。国际指南建议[106]，CMV激活预防应从移植后28 d内启动，持续至移植后100 d。对于cGVHD肺部病变患者，可考虑适当延长疗程或重新启动CMV激活预防，应用至免疫抑制剂减量。更昔洛韦/缬更昔洛韦作为传统抗CMV药物，是目前临床上较常用的一线药物[104],[107]。有研究表明[108]，allo-HSCT后CMV血清学阳性的患者预防性使用来特莫韦能够显著降低有临床意义的CMV感染率，且来特莫韦也获得美国移植与细胞治疗学会（ASTCT）国际指南[106]的推荐。

**临床问题21：**对于确诊cGVHD肺部病变的患者，是否推荐进行呼吸康复及如何进行呼吸康复？

**推荐意见21：**推荐cGVHD肺部病变患者戒烟，在专业医师的指导下进行呼吸康复，包括系统的呼吸训练、吸气和呼气肌力训练、呼吸道清理，以及有氧运动和抗阻运动等；同时指导患者自我监测和管理，加强对自身活动耐力变化及呼吸困难相关症状的识别；对于严重慢性静息性低氧血症患者，建议长期氧疗（推荐强度B，证据等级2b）。

证据总结：呼吸康复已被证实可以改善呼吸困难症状，并可提高患者的运动耐量。目前国内外共识均提出支持性干预措施包括戒烟、呼吸康复、在严重慢性静息性低氧血症情况下长期氧疗等对cGVHD-BOS患者有益[7],[35]。临床研究证实，呼吸康复（包括呼吸训练、有氧运动、力量训练等）可以改善BOS患者的肺活量、呼吸肌力量、活动耐量及呼吸困难主观症状，同时还可改善患者6分钟步行距离及SF-36身体功能评分[109]–[110]。

九、总结与展望

cGVHD肺部病变是allo-HSCT的一种严重并发症，严重影响患者预后和生活质量。cGVHD肺部病变正逐步进展为多种临床表现与体征，目前仍以BOS和ILD表现为主。cGVHD肺部病变起病隐匿，早期诊断困难，且由于其进行性加重的特点及治疗选择有限，与较高的发病率和死亡率有关，因此重视肺部cGVHD的诊治具有重要意义。cGVHD肺部病变作为需要长期监测与干预的慢性疾病，实施多学科综合诊治模式，构建cGVHD慢性病全程化管理至关重要。

肺功能检查、影像学评估与生物标志物检测有助于早期识别疾病进展风险，及时进行抢先干预。cGVHD肺部病变的治疗方法包括口服阿奇霉素、吸入疗法和FAM方案等，新型靶向药物（贝舒地尔、芦可替尼等）、其他药物（吡非尼酮、尼达尼布）、MSC等在改善肺功能方面均有一定效果，但目前循证证据非常有限，仍需通过大规模、高质量的临床试验进一步验证。对于病情严重且对药物治疗无反应的患者，肺移植仍是一种可行的选择。感染预防以降低与免疫抑制相关的风险和支持性干预措施如呼吸康复在cGVHD肺部病变的管理中也同样重要。未来的研究应侧重于持续推进统一的cGVHD肺部病变精准分期分级系统，并推动建立肺功能变化、影像学特征及标志物为支撑的组合评价系统，联合cGVHD肺部病变患者报告结局及生存质量分析调研，有助于推动cGVHD肺部病变的个性化诊疗。

本共识临床问题及推荐意见汇总见附表1（**扫描本文首页二维码查阅**）。

## Supplementary Material


